# Expanding the discourse: a critical analysis of hip-hop feminism in the era of globalization

**DOI:** 10.3389/fsoc.2025.1525981

**Published:** 2025-03-24

**Authors:** Xi Ling, Yuanyuan Chen, Xuanmin Zhu

**Affiliations:** ^1^Physical Education Department, Fuzhou Institute of Technology, Fuzhou, Fujian, China; ^2^Faculty of Education, Silpakorn University, Sanam Chandra Palace Campus, Nakhon Pathom, Thailand

**Keywords:** hip-hop feminism, hip-hop feminist, hip-hop, feminism, feminist

## Abstract

The emergence of hip-hop feminism as a cultural, intellectual, and political movement has captured global attention in recent years, highlighting its significance in contemporary discourse. This review aims to re-examine the definition of hip-hop feminism, particularly in the context of globalization and digital media, and to explore its impact on challenging gender stereotypes and enhancing women’s discursive power. Through a critical review and analysis of existing literature, this review reveals the deficiencies in the definition of hip-hop feminism and proposes updated and expanded definitions that more comprehensively reflect its global and cross-cultural dimensions. The findings suggest that hip-hop feminism encompasses a broader spectrum of activities and influences. It not only addresses the lives and perceptions of black women but also extends to performative and ethnographic studies, engages actively in digital platforms such as blogs and online communities, and facilitates new avenues for women’s political engagement and social activism on a global scale. This review poses critical questions to address potential gaps in the existing literature, thereby mitigating the risk of incomplete or biased definitions of hip-hop feminism. It further proposes three robust theoretical frameworks to comprehensively define hip-hop feminism.

## Introduction

Hip-hop, which initially emerged as a cultural movement in the Bronx, New York, has transcended its local origins to evolve into a global phenomenon (Ling et al., 2025; Yang et al., 2022). The international proliferation of hip-hop can be ascribed to its inherent adaptability and its capacity to resonate with a wide array of cultural contexts. In recent years, there has been a growing interest in research on the use of hip-hop feminism to help women challenge gender stereotypes and enhance female discourse. Many studies have demonstrated that hip-hop feminism can help women reshape their personal image (Cooper, 2017; Morgan, 2000; Parker, 2023), inspire potential and self-confidence (Durham, 2018), and elevate the image of female groups in the public eye and status (Collins, 2022; Pough, 2004). Hip-hop feminism scholars Aisha Durham et al. define hip-hop feminism as the following:

*Hip-hop feminism as a cultural, intellectual, and political movement grounded in the situated knowledge of women of color from the post–civil rights or hip-hop generation who recognize culture as a pivotal site for political intervention to challenge, resist, and mobilize collectives to dismantle systems of exploitation* (Durham et al., 2013, p. 721).

In their study, they adopted a review and analysis of the literature to describe the evolution of hip-hop feminism and the current state of research (Durham et al., 2013). This study leads the authors to conclude that hip-hop feminism focuses on black women as a culturally, intellectually and politically integrated movement that explores the lives and perceptions of women in the hip-hop generation. At the same time, the field expands into performativity and ethnographic studies, is active in blogs and online communities, and provides new platforms for women’s political participation and social activism (Durham et al., 2013, p. 732).

Prior definitions, primarily centered on the African American cultural context, have often overlooked globalization’s significant influence on hip-hop feminism’s evolution and diffusion, constraining our comprehension of its varied cultural expressions (America, 2014; Moji, 2018). The phenomenon has become globally pervasive, particularly in regions like Africa, Latin America, and Asia, where an increasing number of women articulate feminist ideologies through localized hip-hop art (Baker, 2020; Castro, 2016). Furthermore, the critical role of digital media in modern hip-hop feminism has been largely unexplored, despite the expansion of female artists’ reach through social media and streaming platforms. Additionally, the inclusivity and intersectionality of hip-hop feminism are limited by the insufficient attention given to non-binary gender identities and the LGBTQ+ community (Medina García et al., 2023).

In this context, we felt the need to ask challenging questions to reduce the risk of an incomplete or one-sided definition of hip-hop feminism due to inadequate literature review. These questions include whether the existing literature provides a comprehensive account of hip-hop feminism, how researchers can properly access the literature on the definition of hip-hop feminism, and to what extent do the results of the research meet the requirements of the definition of hip-hop feminism?

In addition to the issue of insufficient literature review, we found that Durham et al. (2013) definition of hip-hop feminism is based on both a partial and ambiguous understanding. A major reason for this is that they lack both an understanding of the definition of hip-hop and the concept of feminism itself. For example, the definition of hip-hop feminism focuses on the “cultural, intellectual, and political movement” (Durham et al., 2013, p. 722) without adequately articulating the core elements of feminism, such as gender equality, rights and bodily autonomy, among other fundamental principles (Alexander, 2006). For example, Durham et al. (2013) overemphasize hip-hop feminism as an “emerging” political movement (p. 722), ignoring the contribution of hip-hop as a central element of hip-hop feminism, and failing to focus on the links and inheritance between feminism and hip-hop feminism. One consequence of failing to adequately explain the definition of feminism is that future hip-hop feminism researchers misunderstand the definition of hip-hop feminism, inadvertently spreading misinformation leading subsequent researchers to misuse inaccurate definitions in their publications, and the resulting series of cognitive misunderstandings will result in a cascade of definitional errors.

While we concur with Durham et al.’s application of hip-hop feminism to enhance women’s discourse, we identify significant challenges in their thesis, categorized as etymological, structural, and contradictory. Notably, Durham et al.’s definition does not stem directly from Morgan (2000), and it omits a nuanced understanding of feminism within the realm of hip-hop feminism. Moreover, their portrayal of hip-hop feminism as a singular cultural movement overlooks the intricacies within hip-hop’s internal structure. The contradictions arise from the broader scope of hip-hop feminism, which extends beyond advocating for female hip-hop artists to include the unification of diverse social groups in the pursuit of equity and justice (Veltre and Hadley, 2012). These issues stem partly from Morgan’s initial vague depiction of hip-hop feminism, which has led to misinterpretations and one-sided understandings among subsequent researchers. This has overshadowed other critical aspects of hip-hop feminism, such as its connection to black feminist traditions, its global reach, and its alignment with black men in advocating for shared rights within the black community (Colley, 2019; Delmar, 2018; Hooks, 2019; Moji, 2018; Peoples, 2008). The uncritical acceptance of these limitations has solidified a narrow trajectory for hip-hop feminist studies, hindering a more comprehensive and diverse comprehension of the field.

In this context, this review poses two questions, the results of which will be used to recharacterize and expand the definition of hip-hop feminism.

Research question 1 (RQ1): What is feminism in the context of hip-hop feminism?

Research question 2 (RQ2): What are the hip-hop connotations in the definition of hip-hop feminism?

Here, we hope to activate a discussion on the definition of hip-hop feminism and to revise and restating those ideas related to feminism in the context of hip-hop feminism. We believe that a definition of hip-hop feminism should include a clear delineation of the domain of the concept to avoid overlap and confusion of terms. It must include an explanation of the term feminism and should clearly articulate the uniqueness of hip-hop in hip-hop feminism, the specific application of feminism within the theoretical framework of hip-hop feminism.

## Methods

Before reviewing hip-hop feminism, we must first clarify identify the core concepts of feminism and define the scope of the discussion of gender egalitarianism in hip-hop culture. While exact definition of what constitutes hip-hop feminism vary, most definitions, at a minimum, at least include advocacy for hip-hop female. As stated in Grant and Booth (2009) opinion, “an effective critical review presents, analyses and synthesizes material from diverse sources” (p. 93). In order to capture as much relevant research as possible, we followed the PRIESMA methodology and conducted a literature search of literature databases (Moher et al., 2015).

### Search strategy

Based on critical feminist theory and globalization theory, the researcher selected several core articles on black women, female expression in the context of globalization, and the impact of hip-hop culture for textual analysis. Critical perspectives were used to gather and analyze the content of existing definitions of hip-hop feminism and to provide an in-depth examination and assessment of the shortcomings of these definitions. The researcher collected literature from the Web of Science and Scopus databases, which are considered to be one of the most widely used resources by researchers (Pranckutė, 2021). Based on critical feminist theory and globalization theory, the researcher selected several core articles on black women, female expression in the context of globalization, and the impact of hip-hop culture for textual analysis. Search methodology terms and fields used to locate articles is detailed in Table 1.

**Table 1:** Search methodology terms and fields used to locate articles.

Search methodology keywords and fields used to locate articles		
Web of science	Search Terms 1	TI = (“Hip-hop” OR “hip-hop” OR “hip hop” OR “Hip hop”)
	Search Terms 2	ALL = (“feminism” OR “feminist”)
Scopus	Search Terms 1	TITLE-ABS-KEY (“Hip-hop” OR “hip-hop” OR “hip hop” OR “Hip hop”)
	Search Terms 2	TITLE-ABS-KEY (“feminism” OR “feminist”)

### Inclusion and exclusion criteria

Table 2 delineates the terminological framework employed in the literature review, alongside the rationale for the inclusion and exclusion of specific sources.

**Table 2:** Inclusion and exclusion criteria.

Inclusion criteria	Exclusion criteria
Investigating the definition of hip-hop feminism.	Book, book chapters, and conference papers
Hip-hop as an expression of hip-hop feminism, e.g., peace, love, unit, having fun; Hip-hop as core values of hip-hop feminism.	The sample does not involve hip-hop female
English language	No hip-hop feminism content involved
Journal articles published	Systematic literature review

**Figure 1:**
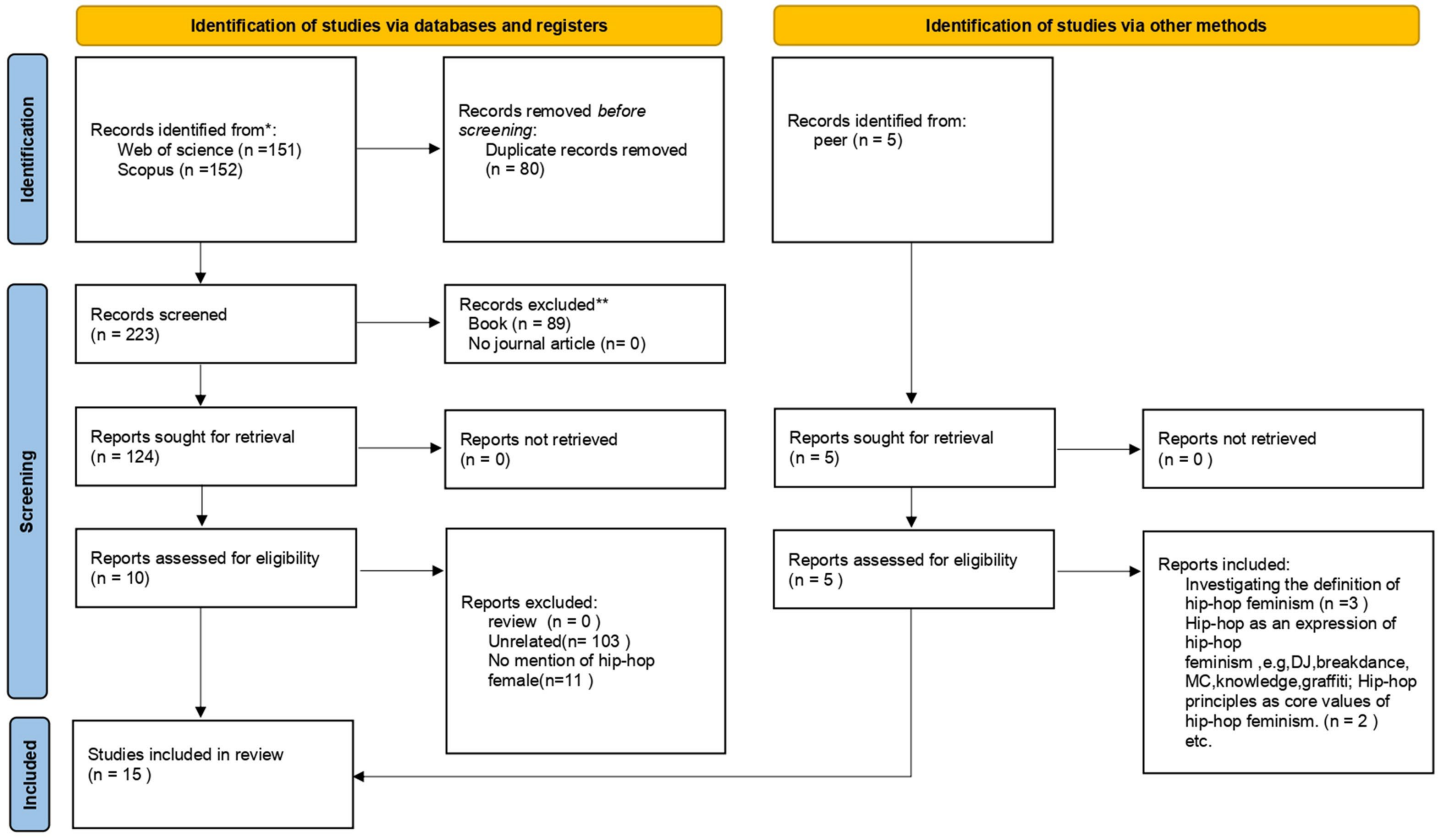
Literature review and incorporation flowchart.

Figure 1 illustrated the literature review and incorporation diagram. This diagram has been modified to detail the various stages of the literature review process involved in identifying and screening peer-reviewed research literature.

## Results

Both searches were carried out on October 16, 2024. The articles underwent a meticulous manual review conducted by a duo of researchers. During this process, 67 documents were deemed redundant and thus discarded, while 89 books and their respective chapters were excluded from further consideration. An additional 11 documents were rejected on the grounds that their titles or abstracts did not pertain to the focal topic of the study. Any discrepancies in the assessment by the two researchers were amicably resolved through collaborative discussion. In an effort to ensure comprehensiveness, Google Scholar was employed as a method of triangulation to ascertain the presence of any further relevant literature in adjacent fields. This strategy led to the identification and subsequent inclusion of 15 additional articles. Upon rigorous screening against the established inclusion criteria, an additional five articles were deemed eligible for inclusion. Ultimately, the data analysis was enriched with the incorporation of 15 articles that met the stringent criteria for this review.

### Data extraction

We have constructed a data extraction table by meticulously extracting pertinent information from the selected articles. The data encompassed in this table includes: full reference citations, the country of origin, the research question or focal point, the sample size and duration of the study, the methodologies and data collection procedures, as well as responses to Research Questions 1 and 2 (See Table 3 for details).

**Table 3:** Detailed summary of result.

No	Full reference	Country	Research question or focus	Sample and research duration	Methods and data collection	RQ1 and RQ2
1	Durham et al. (2013)	USA	This text explores the rise and development of hip-hop feminism, which is a cultural, intellectual, and political movement.	None	None	**RQ1:** Feminism insisting on centering women of color in the analysis. **RQ2:** In hip-hop feminism, “hip-hop” is both a generational marker and a cultural art form.
2	Parker (2023)	UK	The article mainly focuses on the intersection of hip-hop culture, feminism, and education.	None	None	**RQ1:** Feminism has provided black women with the opportunity to “reshape, reimagine and rebuild themselves, their history, culture and language,” becoming an important carrier for expressing and realizing feminist ideals. **RQ2:** Hip-hop feminism embodies the core principles of hip-hop culture, such as “peace, unity, love, and having fun,” providing a space for marginalized groups to express themselves and find identity, and promoting critical democratic education.
3	Eltantawy and Isaksen (2020)	USA	The article focuses on the American-Syrian artist Mona Haydar, exploring how she combines Islamic feminism and hip-hop feminism for Islamic resistance.	None	The study used a combination of critical rhetoric and interviews with the participants to analyze the content of Haydar’s music videos.	**RQ1:** Feminism emphasizes opposition to male dominance, educating the audience, and empowering women through creative expression. **RQ2:** Creative expressions in hip-hop culture (such as music, graffiti, and breakdance) are used as a medium for hip-hop feminism to resist oppression and express women’s demands.
4	Chew and Mo (2019)	China	The article documented their struggles against gender inequality, and discussed how these inequalities and struggles differ from the situation in the US and research on women in hip-hop music.	None	The article used interviews with b-girls and participant observation to collect data.	**RQ1:** Feminism believes that Black women are using hip-hop to empower themselves, calls for critics to consider the intersectionality of gender and race/class, and envisions a progressive and intersectional gender politics based on hip-hop. **RQ2:** Hip-hop consists of nine elements, but popular and academic discourse tends to use the term “rap” interchangeably with “hip-hop,” which has led to a popular understanding of hip-hop that is overly skewed toward rap music.
5	Payne (2024)	USA	This study explores how Black girls reflect, negotiate, reject, and connect their racial and gender identities through hip-hop culture (such as rap music, dance, graffiti/art).	6 Black girls aged 12 from the southern United States who participated in extracurricular programs focused on hip-hop.	Semi-structured interviews. Thematic analysis.	**RQ1:** Feminism emphasizes redefining Black women’s identities by identifying contradictions in identity expression and construction (“doing the gray”) and subverting racist and sexist ideological rhetoric (“bringing the ruckus”). **RQ2:** Hip-hop culture is a cultural phenomenon related to the lives, experiences, and cultures of Black and minority youth. Hip-hop includes 5 elements: MC (rapping), DJ, graffiti art, break dancing, and self-awareness.
6	Johnson (2021)	Canada	How does the performance of Black women in Southern hip-hop culture geographically locate the South, thereby expanding the narrative of “dirty South” hip-hop?	None	Literature review method. Analyzing literature related to Southern hip-hop feminism.	**RQ1:** Emphasizes the creativity and self-expression of Black women and girls in hip-hop culture. Challenges the stereotypical portrayal of Black women as victims in hip-hop culture, emphasizing their agency in sexual expression and body politics. Focuses on the unique experiences and expressions of Black women and girls in Southern hip-hop culture, providing them with more representation and identification. **RQ2:** Musical and cultural traditions, including Southern accents, laid-back atmosphere, bass-heavy beats, simple call-and-response lyrics, booty/twerk movements, and sexually-themed chants.
7	Durham (2009)	USA	This article explores how race, gender, and class intersect in the lived experiences and commodification practices of African Americans within hip-hop culture.	None	Autobiographical ethnographic method. Analysis of the documentary “Beyond Beats and Rhymes.”	**RQ1:** Feminism is a sociocultural, intellectual, and political movement grounded in the knowledge of the conditions of women of color, aiming to challenge, resist, and mobilize through cultural intervention to dismantle exploitative systems. **RQ2:** Hip-hop is defined as a “home” space of culture and knowledge, where the author, as an “insider-outsider,” seeks self-identification.
8	Halliday and Brown (2018)	USA	The article explores how Nicki Minaj and Beyoncé’s collaborative song “Feeling Myself” has become an anthem of “Black Girl Magic” as a form of political empowerment for Black women.	The author himself. Middle-aged. Duration is not clearly stated.	Focus group discussions Collect feedback from young Black women on the “Feeling Myself” song and music video.	**RQ1:** As an extension of Black feminism, feminism emphasizes the importance of Black women expressing themselves and seeking pleasure in contradictory spaces. **RQ2:** Hip-hop feminism views hip-hop culture as a space for Black women to express their racial and ethnic identities, and to critique racism.
9	Moore (2023)	USA	The article explores hip-hop feminism theory and practice, as well as the expression and status of Black women in hip-hop culture.	None	None	**RQ1:** Feminism is a Black women-centered feminist theory and practice that aims to challenge the stereotypes and biases against Black women in mainstream culture, and to express the experiences and demands of Black women. **RQ2:** Hip-hop has a mystical and transcendent connotation in the context of hip-hop feminism.
10	Opara (2024)	USA	This paper explores the status and experiences of African American women in the hip-hop music industry.	None	Various methods, including analysis of music videos, interviews, audience reactions, and references to relevant academic literature.	**RQ1:** Feminism reflects some fundamental feminist principles, such as challenging gender inequality and racial discrimination, and providing a space for Black women to express themselves. **RQ2:** Hip-hop music provides a platform for Black women to express themselves and promote solidarity, which is related to the core principles of hip-hop culture, such as “peace, unity, love.”
11	Lindsey (2015)	USA	This paper aims to summarize and analyze several key hip-hop feminist theoretical interventions.	None	Literature review and analysis.	**RQ1:** Feminism is a feminism centered on the experiences and perspectives of Black and women of color. **RQ2:** In hip-hop feminism, hip-hop represents a cultural tradition shaped by Black and women of color, reflecting their experiences, perspectives, and positions.
12	Carney et al. (2016)	USA	This article explores how Black girls express their sexual knowledge and bodily experiences through hip-hop music and culture, despite facing moral panic and opposition.	None	The article employs methods such as engaging with Black girls’ community art outreach work, analyzing music created by Black girls, and participating in hip-hop and feminist debates.	**RQ1:** Black girls’ expression of their sexual knowledge and autonomy through hip-hop music and culture reflects feminist principles such as sexual self-determination and self-actualization. **RQ2:** Hip-hop culture provides Black girls with an unpoliced, creative space to express their sexuality.
13	Craig and Kynard (2017)	USA	This paper explores the contributions of female hip-hop DJs, highlighting their roles as cultural producers, sound theorists, and rhetorical innovators in a male-dominated industry.	Six pioneering female DJs, spanning over 30 years of hip-hop history.	Semi-structured interviews. Thematic analysis.	**RQ1:** Feminism rejects equating academic distance with the ability to construct knowledge, and instead respects diverse voices. **RQ2:** Hip-hop is viewed as a way of producing knowledge and engaging in political practice, manifested in technical practices such as sound collage and remixing.
14	Gardner (2014)	USA	This paper aims to critically review the literature on Transnational Hip Hop Feminisms (THHF) and explore the relevant discourses of artists and activists from around the world.	None	Literature review method	**RQ1:** Feminism emphasizes the important role of women in various aspects of hip hop culture (such as music production, rapping, breakdancing, and graffiti), and utilizes hip hop culture to promote social justice and educational reform. Hip hop feminist artists express in their works the values of women’s sexual liberation, subjectivity, respect, and celebration of the female body, reflecting feminist principles. **RQ2:** Hip hop, as a transnational cultural art form and practice, is not limited by its creativity, politics, genres, multimodality, and cultural hybridity.
15	Saunders (2016)	USA	By taking a transnational approach, US-based advocates can enhance the possibilities of hip-hop feminism as a politics of solidarity and mutual empowerment.	The author conducted participant observation and interviews in Havana, Cuba from 1998 to 2010, and in São Paulo, Brazil from 2008 to 2013.	Participant observation and interview methods.	**RQ1:** “Peace, unity, love, and having fun.” principles are consistent with fundamental feminist principles such as solidarity and mutual empowerment. **RQ2:** Hip-hop is defined as a “street-based street culture,” with a language and mode of expression that has a mission of directly conveying information, including visual arts, bodily expression, rhythm, rhyme, and poetry.

## Research Question 1 (RQ1): What is feminism in the context of hip-hop feminism?

Feminism in the context of hip-hop feminism reflects a variety of fundamental feminist principles, most notably intersectionality, which is the examination of how different forms of oppression such as racism, sexism, and classism interrelate and impact the experiences of individuals, particularly women of color (Durham et al., 2013). This approach insists on centering women of color in the analysis, recognizing their unique experiences and challenges. Additionally, hip-hop feminism emphasizes empowerment and resistance against male dominance, as well as the importance of creative expression for educating audiences and empowering women (Eltantawy and Isaksen, 2020; Johnson, 2021). It also focuses on redefining Black women’s identities by challenging racist and sexist ideologies (Payne, 2024), and it calls for a progressive and intersectional gender politics that is rooted in the experiences and perspectives of Black women and women of color (Chew and Mo, 2019; Lindsey, 2015).

It is noteworthy that hip-hop feminism has not only demonstrated its influence in Western countries, but also the handling of intersectional issues of race, class, and gender by non-Western hip-hop feminism on a global scale is evident to all. The study by Chew and Mo (2019) serves as a case in point. In Hong Kong, China, Taiwan, China, and Mainland China, female breakdancers, exemplified by informant E, reshape their identities and images through breakdancing, signaling to the public that health is not solely represented by thinness, but also by muscles and strength, and that breakdancing is not an exclusive domain of males (Chew and Mo, 2019). Furthermore, in Japan, female breakdancers also face similar challenges as their (Alexander, 2006; Baer, 2018) Chinese counterparts in competitions, such as the need to exhibit support and respect rather than aggression (Dixon, 2016); In South Africa, female hip-hop artists express their resistance to racism and gender inequality through music and dance (Berggren, 2014); Hip-hop feminism in Latin America also focuses on the rights of transgender and LGBTQ+ communities, promoting broader gender equality (Medina García et al., 2023).

### Definitions and descriptions of feminism in the context of hip-hop feminism

A number of articles indicate an intention to focus on the concept of feminism in the context of hip-hop feminism through titles, keywords or abstracts (Alexander, 2006; Baer, 2018). For example, feminism is defined as a social and political movement aimed at advancing gender equality and challenging patriarchal power structures that marginalize and oppress women (Bhandari, 2024). Women should have equal rights and status and through participation and self-identification, women can rebel against entrenched cultural misogyny (Parker, 2023), feminism is centered on resisting dominant ideologies and creating spaces for critical dialogue (Eltantawy and Isaksen, 2020; Halliday and Brown, 2018), feminism should not only consider gender, but also focus on broader social structural issues such as race, class, and other intersectional oppressions (Gardner, 2014). Regardless of whether these papers focus on multiple definitions or a single definition, each paper included in the review reflects the following claims about feminism in the context of hip-hop feminism:

1. Feminism is a global social movement that advocates for the empowerment of women with equal social, economic and political rights through changes in social structures, cultural ideologies and legal frameworks.
2. Feminism is a dynamic and evolving theory and practice that promotes the multidimensional emancipation of women by critiquing the multiple oppressive structures of race, gender and class in the dominant culture.

As can be seen, Feminism is characterized as a diverse and complex field that has evolved over time, with numerous theories and movements emerging in response to changing social and political contexts (Bhandari, 2023). Beyond that, we illustrate these claims here by citing a series of explanations from articles from a variety of backgrounds. Regarding the first intellectual statement, Cott (1987) delved into the term’s historical and linguistic trajectory, suggesting that its usage in the late 19th century signified both a commitment to women’s emancipation and the broader implications of gender equality. For example, Cott (1987) noted.

*Feminism did not only articulate the goals of women seeking the right to vote but also encompassed a larger vision of transforming societal norms around gender. It was in the early 20th century that the word ‘feminism’ came to represent this wider spectrum of advocacy for women’s equal participation in all spheres of life* (p. 13).

According to Cott (1987), the term “feminism” seems to go beyond a single legal reform and aims at a comprehensive change in gender-related social norms to ensure women’s equal participation and opportunities, reshaping institutions, values, and relationships to Feminism is about reshaping institutions, values and relationships to reflect gender equality. Indeed, there have long been authoritative voices in the field of feminism calling for gender equality, according to Sotiriadou and de Haan (2019), “gender equity denotes fairness and justice in the distribution of gender equity denotes fairness and justice in the distribution of opportunities, responsibilities, and benefits available to men and women, and the strategies and processes used to achieve gender equality” (p. 3). We fully agree with this view. Feminism is about reshaping society to dismantle entrenched patriarchal norms, with its purpose being to enable women’s full, equal participation across all spheres of life (Knowledge Claim 1). Feminism emphasizes the importance of gender in shaping the structure of society, yet this definition does not exclude the influence of other important factors such as race, class, and sexual orientation. Colley (2019) explains Feminism is not only about gender equality, but about the pursuit of justice and fairness in a wide range of social, economic, and cultural contexts. For instance, Colley (2019) wrote:

*I define feminism as a societal structure that “argues the centrality of gender in the shaping of our consciousness, skills, and institutions as well as in the distribution of power and privilege”* (p. 430).

In fact, Lauren Colley’s view also responds to the fact that feminism is not only concerned with gender equality, but also with the ways in which other social injustices are intertwined and seeks justice from a holistic search for justice, such as intersectionality of race, class, and sexual orientation. That is, if researchers match the terms “hip-hop” and “feminism” to form “hip-hop feminism,” they should consider feminism’s feature that not only addresses gender differences, but broadly encompasses race, class, culture, and geographic contexts of intersectional oppression (Knowledge Claim 2).

To this end, what should hip-hop feminism consist of? For example, resistance to Intersectionality oppression, cultural critique and engagement, empowerment and agency, sexuality and body politics, social activism and advocacy, reclaiming hip-hop as a space for feminist expression, and critique of mainstream feminism. Parker (2023), in examining the relationship between hip-hop feminism and feminism, revealed the inherited relationship between the two and that hip-hop feminism develops new elements, for example, using hip-hop culture as a resource to address the inherent misogyny faced by young black women. It is believed to fosters “participation, reaction” and “self-identification” (p. 3). Furthermore, according to Eltantawy and Isaksen (2020)’s study, hip-hop feminism inherits several foundational elements of traditional feminism, including the resistance to dominant ideologies through theory-building, the creation of spaces for critical dialogue, and the emphasis on the performativity of identity. In addition, hip-hop feminism innovates by embracing ambiguity and contradictions, infusing broader transnational feminist possibilities, and adopting a “percussive” feminism that allows for the creativity emerging from the tension between competing political and cultural projects.

Then, to sum up, the first point we make about the literature is that the term “feminism in the context of hip-hop feminism” seems to be closely associated with the following keywords: resistance to Intersectionality oppression, Cultural Critique and Engagement. The aim is to redefine the position and voice of women in this hip-hop culture. These keywords correct the perspective of Durham et al. that confines feminism to a specific cultural context, emphasizing its global engagement and systemic emancipatory implications (Carney et al., 2016; Craig and Kynard, 2017; Moore, 2023). In contrast to Durham et al.’s more narrow definition, hip-hop feminism emphasizes resistance through culture, art, and social movements (e.g., hip-hop and Afrobeats) as a tool for resisting oppression, breaking down single-gender perspectives and focusing on intersectionality and cultural diversity (Gardner, 2014; Payne, 2024). It is not limited to a single culture or country, but instead expands globally, emphasizing the diversity of female resistance in different contexts.

### Research Question 2 (RQ2): What are the connotations of hip-hop in the definition of hip-hop feminism?

In the context of hip-hop feminism, “hip-hop” carries connotations that extend beyond the musical genre to include a generational marker and a cultural art form (Durham et al., 2013). Hip-hop, as a cultural space, provides a platform for marginalized groups to express themselves and find identity. It is characterized by core principles such as peace, unity, love, and having fun, which are integral to both hip-hop culture and hip-hop feminism (Parker, 2023). It is also viewed as a cultural tradition shaped by Black and women of color, reflecting their experiences and perspectives (Lindsey, 2015). Hip-hop culture, with its elements such as music, dance, graffiti, and breakdancing, serves as a medium for resistance against oppression and a platform for expressing women’s demands (Eltantawy and Isaksen, 2020). Furthermore, hip-hop is recognized as a transnational cultural art form and practice that is not limited by its creativity, politics, genres, multimodality, and cultural hybridity (Gardner, 2014), providing a platform for black women to express themselves and promote solidarity, which aligns with the core principles of hip-hop culture, such as “peace, unity, love” (Opara, 2024).

### Definitions and descriptions of hip-hop in the context of hip-hop feminism

As mentioned in the methodology section, in order to accurately interpret the concept of hip hop feminism, we need to consider the concept of hip-hop itself in addition to the concept of feminism. In fact, we share the conviction of authors such as Pough (2007) and Durham et al. (2013) that no matter what terminology researchers use when talking about hip-hop feminism, hip-hop is a key part of the definition. Hip-hop principles, as a central component of hip-hop connotations, are considered essential in hip-hop feminism (Wei et al., 2022; Yang et al., 2022). In examining how the principles of hip-hop are embedded in hip-hop feminism, four themes emerge that align these values with the movement’s core focus on empowerment and resistance. As described by Yang and his colleagues, the principals of hip-hop are, “Peace, unity, love, having fun.” (Wei et al., 2022, p. 6) Hip-hop feminism, while rooted in feminist critique and activism, draws heavily from the cultural ethos of hip-hop, which itself embodies these ideals.

#### Peace

Peace means to stop fighting and keep peace (Chang, 2001). In Payne (2024)’s study, participants Evie and Mariah gained a sense of freedom and relaxation through hip-hop music and culture, thus relieving stress and anxiety in their lives. This reflects the idea of “peace” in hip-hop feminism, which is to seek inner peace and self-expression through hip-hop. Hip-hop feminism focuses on the unique experiences and expressions of black women and girls in Southern hip-hop culture, providing them with more representation and identification, thereby promoting “peace” (Johnson, 2021). In addition, Parker (2023) stated that hip-hop culture provides opportunities for marginalized groups to claim, reimagine and reconstruct themselves, their histories, cultures and languages (p. 5). It provides space for self-expression and identity for the disadvantaged, and reduces conflict and confrontation, which embodies the “Peace” principle of hip-hop.

“Peace” fostering an environment of solidarity, mutual respect, and non-aggression, which allows for the empowerment and expression of women from various marginalized backgrounds (Opara, 2024; Saunders, 2016). It is a principle that guides the movement’s approach to social change and community building.

#### Unity

The focus on collective empowerment and unity is central to both hip-hop and hip-hop feminism. Hip-hop feminists, as Parker (2023) elaborates, use the movement to foster “participation, response, and self-identification” among young black women (p. 3). The unifying aspect of hip-hop principles is mirrored in this collective empowerment, which encourages solidarity among women of color in their fight against cultural misogyny. This unity is also reflected in the transnational feminist possibilities that expand the boundaries of hip-hop feminism (Eltantawy and Isaksen, 2020). Unity is widely spread among female hip-hop artists across the globe. According to Eltantawy and Isaksen (2020), women seek a sense of belonging and identity by joining Muslim organizations that provide services to the community and embody the spirit of “unity.” In addition, the process of spreading and exchanging hip-hop culture around the world, especially in the black august hip-hop project, where American hip-hop artists and South African hip-hop artists seek transnational exchanges and dialogues through hip-hop culture despite differences and conflicts, reflects the principle of “solidarity” (Gardner, 2014).

Notably, Pough (2004), Morgan (2000), and Durham et al. (2013) all emphasize the importance of cross-gender dialogue within hip-hop feminism, advocating collaboration between Black women and men to resist both gender and racial oppression. Pough (2004) highlights how feminist critiques of misogyny in hip-hop seek alliances with Black male artists to challenge systemic racism. Morgan (2000), meanwhile, promotes a feminism of complexity that transcends gender conflicts, and Durham et al. (2013) underscore the need for solidarity in cultural practices aimed at racial justice.

“Unity” represents the active formation of solidarity among women within the genre, creating a collective front against systemic oppression and marginalization. It is a dynamic process that involves mutual support, shared resistance, and the amplification of women’s voices within and beyond the hip-hop community.

#### Love

The theme of love, particularly self-love and love for one’s community, is also central to hip-hop feminism (Coleman et al., 2016). Morgan (2000) highlighted that hip-hop feminism encourages black women to develop a feminism that reflects their complex relationship with hip-hop principles, one that resists harmful stereotypes and celebrates their identity. Halliday and Brown (2018) state that Nicki Minaj and Beyoncé’s collaboration represents themes of “self-confidence, women’s empowerment, and political messaging” that relate to the principle of “love” in hip-hop culture (p. 223), reflecting the desire of black women to express their self-identity and empowerment through music. This embrace of self-love and the celebration of identity is a form of resistance to the negative imagery often perpetuated by mainstream media (Saunders, 2016). In addition, hip-hop culture provides space for self-expression and identity for marginalized groups, which can be seen as an expression of “love” that respects and understands the voices and experiences of different groups (Parker, 2023).

“Love” is affirmation of self-worth, the celebration of one’s identity, and the cultivation of a supportive community. It is a principle that encourages solidarity, mutual respect, and the collective promotion of well-being and happiness among women of color within the hip-hop community.

#### Having fun

The notion of “having fun” is closely tied to self-expression and joy in hip-hop culture, and this translates into hip-hop feminism as well. For example, Gardner (2014) emphasizes that many hip-hop feminists use the culture to celebrate the female body and sexual empowerment, often through breakdance, music, and other forms of artistic expression. This highlights the joy and creativity that hip-hop feminism fosters, where women can express themselves freely and enjoy the process of reclaiming their narratives through hip-hop (Saunders, 2016). It is worth noting that creative ways such as music, graffiti and breakdance in hip-hop culture are often seen as a source of having fun (Ling et al., 2023; Wei et al., 2022). The hip-hop movement originated from youth in poor urban areas who expressed themselves through creative ways such as music, graffiti and dance (Eltantawy and Isaksen, 2020), relieving environmental stress and anxiety (Payne, 2024). In addition, through hip-hop dance, black women and girls are able to express themselves and experience joy and pleasure (Johnson, 2021), which exemplifies the hip-hop culture’s principle of “having fun.”

“Having fun” within hip-hop feminism is about the liberation and joy that comes from engaging in the cultural practices of hip-hop, such as music, dance, and visual arts. It is a principle that encourages women to find pleasure in their own expressions and to celebrate their identities within a community that values creativity and enjoyment. This aspect of hip-hop feminism contributes to the movement’s ability to foster positive experiences and resilience in the face of societal challenges.

Hip-hop feminism embodies the foundational values of hip-hop culture—peace, unity, love, and enjoyment—utilizing these principles to establish empowering spaces for women of color that foster self-expression and communal support. This movement actively confronts oppressive structures while celebrating joy and collective strength. The convergence of hip-hop ethos and feminist ideology within this framework underscores a commitment to equality, empowerment, and intersectionality. By advocating for gender equity through the lens of hip-hop’s core values, hip-hop feminism challenges patriarchal, racial, and class-based oppressions.

### Possible explanations for the definition of hip-hop feminism

This section is intended to be an updated, expanded definition of hip-hop feminism that encompasses two key aspects of hip-hop feminism in context, the use of hip-hop and the concept of feminism itself. Before we present interpretations that might define hip-hop feminism, we pause here to review the definition of feminism mentioned in section one.

According to the previous two sections, we summarize the key term hip-hop is used to define the concept of hip-hop feminism in the following way:

1. Peace is not just about individual harmony with the outside world, but collective action to break down oppressive structures.
2. Unit emphasizes confronting gender and racial injustice through collective power.
3. Love is a core concept of self-care and mutual support in hip-hop feminism.
4. Having fun reflects women’s creative freedom and right to express pleasure through hip-hop culture.

The key elements hip-hop feminism literature included in this review hip-hop are often interpreted as the five elements of hip-hop (Chew and Mo, 2019; Gardner, 2014; Payne, 2024), for example, MC, DJ, graffiti, breakdance, and knowledge (Chang, 2007; Rose, 2008; Wei et al., 2023). However, many researchers have made it clear that an understanding of hip-hop should not be limited to manifestations; rather, hip-hop should not ignore the social, political, and cultural significance of hip-hop (Parker, 2023; Saunders, 2016). To this end, we describe the definition of hip-hop as follows.

1. Hip-hop is not only an art form, but also a cultural movement that embraces the core values of “peace, unity, love and having fun.” It promotes social solidarity through music, dance, and community activism, especially the search for common ground amidst racial, class, and cultural differences.
2. Hip-hop is a tool for individual and community empowerment. It encourages self-confidence through self-expression, creativity, and interaction, especially for individuals from marginalized groups. Individuals build new identities and find a sense of self-worth through the elements of hip-hop and bring this sense of empowerment back to their communities, resulting in positive social change.

In addition, in the feminist literature included in this review, the term feminism is often interpreted as a social theory and political movement with gender relations as a central topic of political study (D'ignazio and Klein, 2023; Delmar, 2018), but many researchers have made it clear that an understanding of feminism should not be limited to the female population. For example, Hooks (2000) defined feminism as a movement to end sexism, gender exploitation and oppression. This means that feminism is not only concerned with the rights and interests of women, but also opposes any form of sexism, including those that negatively affect men (Hooks, 2019).

Based on a synthesis of the literature, it is evident that hip-hop feminism has evolved beyond its initial manifestation as an exclusive expression of Black female hip-hop artists, gradually transforming into a global movement for women’s empowerment, particularly providing a platform for women of color to articulate their voices and experiences. This evolution underscores that hip-hop feminism is not confined to specific racial or cultural contexts but is dedicated to empowering marginalized women worldwide, enabling them to express their struggles and contemplations on gender, race, and social injustice through elements of hip-hop culture, such as music, dance, and graffiti. In addition, the broadened conception of hip-hop feminism continues to focus on the rights of Black women while also embracing a diversity of women’s experiences, especially those of women from other cultural backgrounds, such as Latina, Asian, and Indigenous women (Chew and Mo, 2019; Eltantawy and Isaksen, 2020; Moji, 2018; Opara, 2024). It offers a shared space for women of different backgrounds globally to challenge gender inequality, racism, and class oppression through hip-hop culture.

From these perspectives, we delineate the potential criteria for the conceptualization of hip-hop feminism. The examples provided meticulously define the key terms integral to the definition of hip-hop feminism, specifically the concepts of hip-hop and feminism. We offer three definitional templates for hip-hop feminism:

1  Hip-hop feminism is a multifaceted movement at the nexus of cultural expression and social activism. It empowers women of color by reclaiming hip-hop spaces, challenging misogyny, and advocating for gender equality. This movement leverages the principles of peace, unity, love, and enjoyment inherent in hip-hop to foster solidarity and resistance against systemic oppression.

OR

2  Hip-hop feminism integrates feminist theories with the practices of hip-hop culture, creating a platform for women to assert their identities and narratives. It emphasizes the historical and contemporary contributions of women to hip-hop, critiques the genre’s gender biases, and uses creative expressions like music, dance, and graffiti to spark conversations on power, race, and justice.

OR

3  Hip-hop feminism is a critical framework that deconstructs and reimagines the gender politics within hip-hop. It is an academic and activist approach that centers the experiences of women of color, advocating for their empowerment and full participation in hip-hop culture. This definition highlights the intersectionality of race, gender, class, and sexuality, and calls for a feminist practice that is inclusive, diverse, and deeply rooted in the communal spirit of hip-hop.

The purpose and significance of employing these key terms in formulating options is to ensure that the definitions utilized are comprehensive in terms of the requisite essential information, such as the characteristics of Hip-hop principles and the conceptual knowledge associated with feminism. This template is designed to neither dwell on unnecessary details nor omit any critical elements.

## Conclusion

This review aims to ascertain the definition of hip-hop feminism and to identify the pivotal elements of hip-hop as utilized within the framework of hip-hop feminism through a review of the literature. It is important to clarify that achieving a unified consensus within the academic community regarding the definition of hip-hop feminism is unrealistic, given the multitude of conceptual definitions and divergences that exist concerning what constitutes hip-hop feminism. Herein, we also advocate for future researchers in the field of hip-hop feminism to strive for clarity in their articulation of what is included and what is excluded in their definitions, as the elements and principles of hip-hop are prone to confusion and omission. In addition, based on the viewpoints and core features articulated within the literature encompassed by this study, we introduce an operationalizable theoretical framework designed to codify the definition of hip-hop feminism. The aim of this framework is to articulate the connotations of hip-hop feminism in the most succinct terms, thereby offering scholars in this domain a conceptual framework for reference. However, a limitation of this review lies in its narrow focus on the definitional aspects and an insufficient engagement with black feminism. Likewise, due to the constraints of length, the scope of the database searches conducted for this research was not broadened. Despite these limitations, this review marks the inaugural attempt to revise and update the existing definitions of hip-hop feminism, and the definition has the potential for widespread dissemination.

## Data Availability

The original contributions presented in the study are included in the article/supplementary material, further inquiries can be directed to the corresponding author.
